# Important potential risks not reflected in Japanese drug labeling: implications for risk management plan termination

**DOI:** 10.1007/s40199-026-00661-7

**Published:** 2026-09-18

**Authors:** Natsuko Kameyama, Ayana Ide, Hideki Maeda

**Affiliations:** 1 Regulatory Science, Graduate School of Pharmaceutical Science, Meiji Pharmaceutical University, Tokyo, Japan; 2 CMIC Co., Ltd., Tokyo, Japan; 3 Regulatory Science, Faculty of Pharmacy, Meiji Pharmaceutical University, Tokyo, Japan

**Keywords:** Risk management plan, Potential risk, Drug labeling, Pharmacovigilance, Japanese Pharmaceutical Regulations, Safety concerns

## Abstract

**Background:**

Risk management plans (RMPs) support lifecycle management of drug-related risks. In Japan, however, their use in clinical practice remains limited, and healthcare professionals often rely on the package insert for safety information. After RMP termination at re-examination, healthcare professionals may no longer readily identify unresolved risks absent from the package insert through either the package insert or the Pharmaceuticals and Medical Devices Agency (PMDA) RMP source.

**Objectives:**

We investigated the extent to which important potential risks in RMPs are reflected in the package insert and considered the implications of RMP termination when such risks remain undocumented.

**Methods:**

We analyzed RMPs publicly available as of May 2023 using data from the PMDA website. For each important potential risk, we examined whether it was documented in the package insert and summarized the results descriptively by risk type.

**Results:**

Among 772 important potential risks in 330 RMPs, 134 (17.4%) were not documented in the package insert. Non-documentation proportions were relatively high for “Infections and infestations” (32.7%), “Gastrointestinal disorders” (32.4%), and “Neoplasms benign, malignant and unspecified (incl cysts and polyps)” (28.4%), whereas a relatively low proportion was observed for “Vascular disorders” (2.3%).

**Conclusion:**

Some important potential risks in active Japanese RMPs were not documented in the package insert. This may create gaps in risk communication after RMP termination, although clinical outcomes and patient harm were not assessed. Allowing RMP continuation beyond re-examination when necessary may help maintain awareness of unresolved safety concerns.

**Graphical abstract:**

**Figure:**
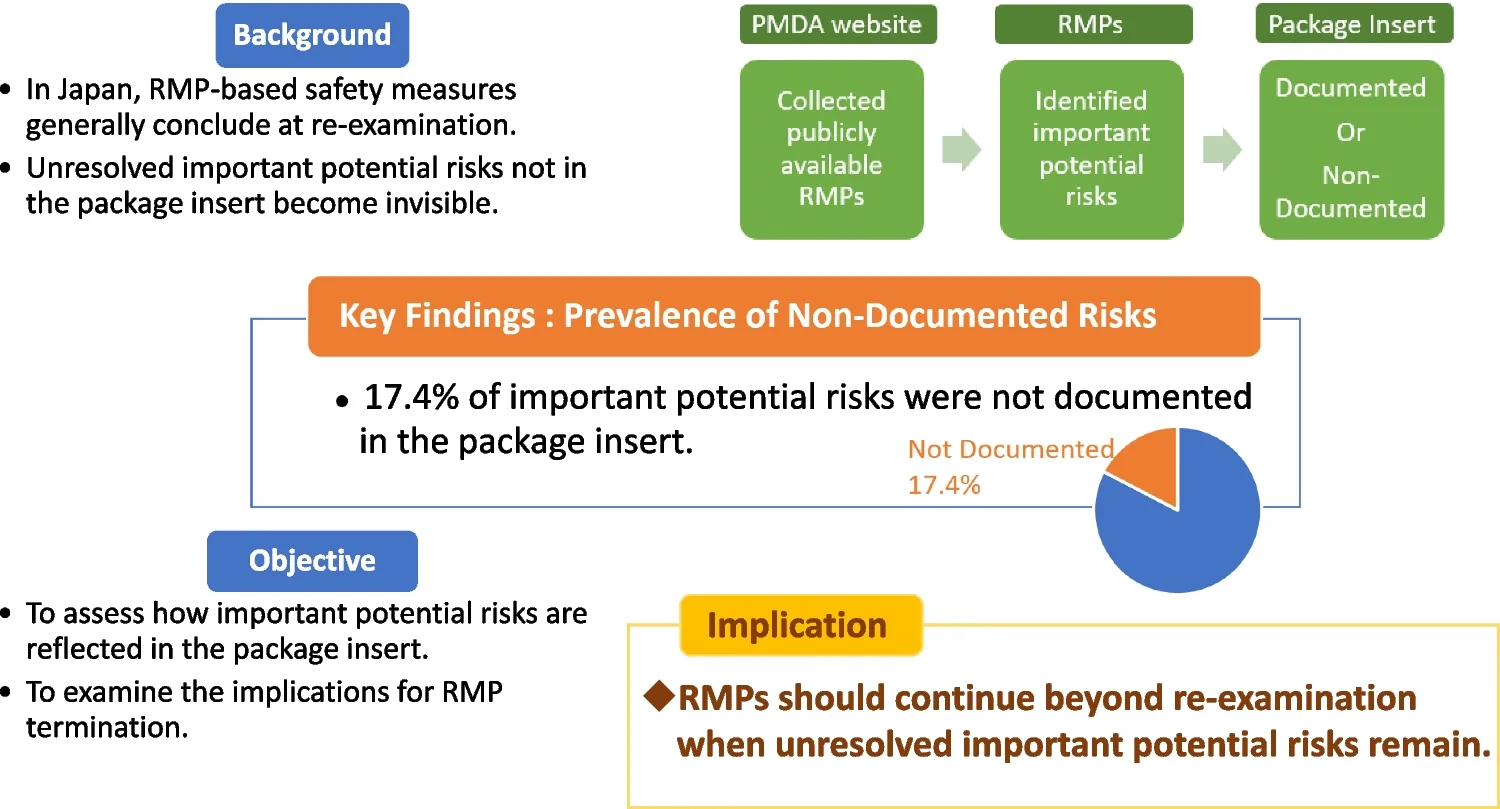

## Introduction

Risk management plans (RMPs) [1, 2] are documents that outline strategies for managing drug-related risks throughout a product’s lifecycle from development to post-marketing. RMPs are used to continuously evaluate and reassess the benefit–risk balance and implement well-defined post-marketing safety measures. RMPs define safety concerns as important identified risks, important potential risks (IPRs), and important missing information, for which pharmacovigilance and risk minimization activities are assigned. Pharmacovigilance activities consist of routine activities such as spontaneous adverse event reporting and additional activities such as post-marketing surveillance. Risk minimization activities include routine measures such as documentation in Japanese drug labeling information, specifically the package insert (PI), and additional activities such as distributing educational materials for healthcare professionals and patients. Within pharmacovigilance [3], the ability to promptly detect changes in the safety profile of drugs and take appropriate action through timely evaluation is essential, and RMPs play key roles in supporting these efforts.

In Japan, the “Guideline on Risk Management Plan” issued in 2012 has applied to new drugs submitted for approval on or after April 1, 2013, making RMP preparation a regulatory requirement [4]. Although more than a decade has passed since RMPs were introduced, their uptake in clinical practice remains limited, making the PI the primary source of safety information for healthcare professionals [5]. However, our previous study, which focused on a limited number of products for which RMPs had been completed at the end of the re-examination period, suggested that some IPRs, as well as the underlying information justifying their designation, were not reflected in the PI. Because most RMPs were also terminated at that point, such undocumented IPRs could no longer be readily identified by healthcare professionals through either the PI or the Pharmaceuticals and Medical Devices Agency (PMDA) RMP source [6]. Although this exploratory finding highlighted a potential concern, the overall extent of the issue remained unclear. As IPRs are often later confirmed as serious adverse reactions [7, 8], clarifying the prevalence of undocumented IPRs is essential. Currently, there is no straightforward method for healthcare professionals to confirm undocumented IPRs after RMP completion.

For these reasons, we comprehensively investigated the documentation of IPRs in PIs, aiming to inform discussions on the appropriateness of RMP completion when undocumented IPRs persist. Rather than demonstrating a direct link between non-documentation and patient harm, our study highlighted a regulatory transparency gap that arises when unresolved IPRs can no longer be readily identified through either the PI or the PMDA RMP source after RMP termination. Although Sawada et al. provided an overview of all active RMPs as of 2023 [9], our study adds further value by investigating the extent to which IPRs are reflected in the PI. Clarifying this issue is important for supporting future improvements in Japan’s relatively new RMP system. Beyond the Japanese context, this issue also has international relevance. Although RMPs were harmonized under the ICH E2E guideline, their implementation and the extent to which safety concerns are reflected in product labeling differ across regions [10]. Therefore, this study comprehensively evaluated how IPRs are documented in the PI and considered the implications for the appropriateness of RMP termination in Japan while also offering insights for international discussions on risk communication and lifecycle risk management.

## Methods

The study targeted RMPs that were active as of May 2023. Because the analysis was conducted in May 2024, RMPs that were revised or removed between June 2023 and May 2024 were excluded, as their original versions were no longer accessible through publicly available PMDA sources. This ensured that the study cohort was based on a consistent and reproducible dataset. RMP information was partially obtained from a database created by Sawada et al. [9] based on the list of active RMPs published on the PMDA website [11]. In that study, robotic process automation tools were used to extract product names, active ingredients, and safety concerns. These safety concerns were mapped by three coders to the Lowest Level Terms of the Medical Dictionary for Regulatory Activities (MedDRA/J) version 26.0 [12] and categorized into System Organ Classes. In the present study, we further reviewed IPRs that had not been assigned a MedDRA code in that database. All 33 of these IPRs concerned drug administration to patients with comorbidities, relevant medical histories, or other specific conditions and were therefore classified separately as “Safety and efficacy in specific populations.” This non-MedDRA category was mutually exclusive with the System Organ Class categories. Additional information not included in the database of Sawada et al., including information on products covered by each RMP and associated additional activities, was obtained from the PMDA’s “Information Search for Prescription Drugs” [13]. From these sources, we obtained information on IPRs and their MedDRA coding, pharmacovigilance activities, risk minimization activities, and therapeutic categories [14] and subsequently constructed an original database.

For all IPRs listed in the target RMPs, we investigated the proportion of IPRs that were not documented in the PI. We defined “documented in the PI” a priori as “PI inclusion” listed as a risk minimization activity in the RMP for that IPR. The documentation status was determined exclusively from the RMP listing rather than from direct PI text review. We adopted this objective operational definition to avoid inconsistent assessments that can arise from subjective visual review of PI text [15]. We validated this indirect classification by directly reviewing a simple random sample of 60 IPRs selected from all 772 IPRs and examining the most recent PI texts. Two reviewers independently inspected the PIs, and discrepancies were resolved by consensus. Agreement, sensitivity, specificity, and Cohen’s κ were calculated by treating “non-PI documentation” as the positive class. The misclassification-corrected estimate was calculated using the Rogan–Gladen estimator based on the sensitivity and specificity obtained from the validation sample. We further classified IPRs by risk type and examined the proportion of IPRs not documented in the PI in each subgroup. For IPRs documented in the PI, we reviewed which sections of the PI [16] were designated for risk minimization activities. For IPRs not documented in the PI, we identified the reasons for non-inclusion in the PI.

This study was reported in accordance with the Strengthening the Reporting of Observational Studies in Epidemiology (STROBE) guideline for cross-sectional studies [17]. As the primary objective, this study descriptively investigated the extent to which IPRs are documented in the PI. Risk-type-specific results were also summarized descriptively. The 95% confidence interval for the overall proportion was calculated using the Wilson score method.

## Results

**Fig. 1:**
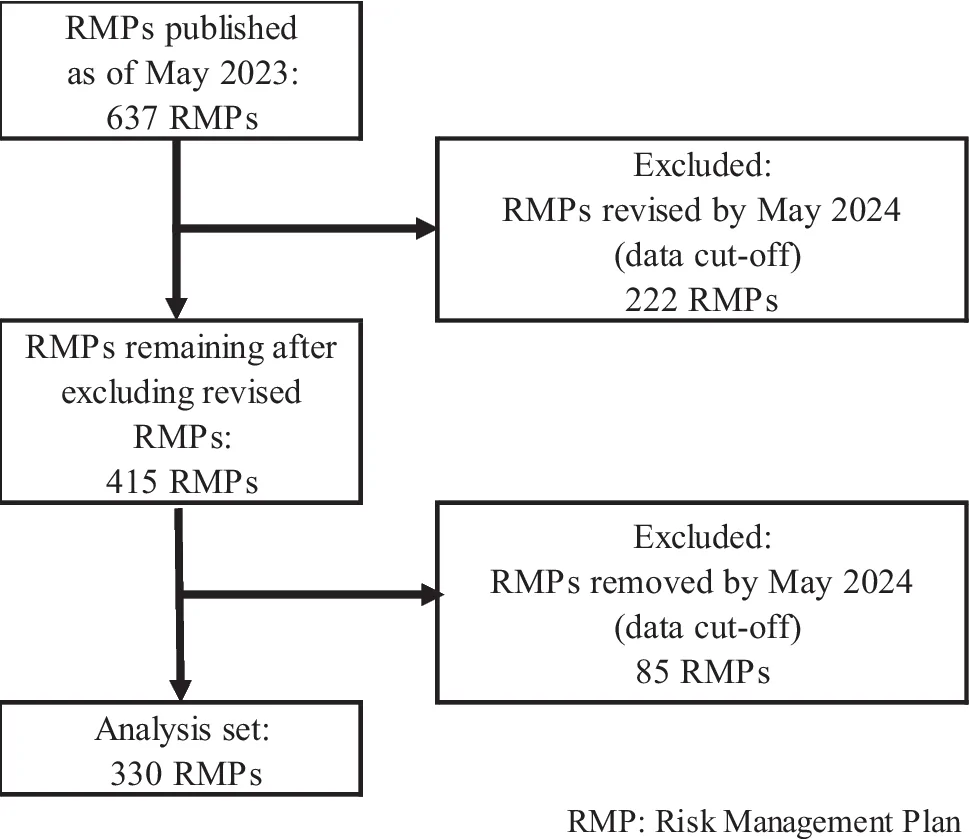
Inclusion and exclusion criteria

Among the 637 publicly available RMPs as of May 2023, 222 and 85 RMPs that had been revised and removed between June 2023 and May 2024 (the database cutoff date), respectively, were excluded because their original versions were no longer accessible through publicly available PMDA sources at the time of analysis, leaving 330 RMPs for analysis (Fig. 1). In total, 772 IPRs were identified from the 330 RMPs. Therapeutic categories of these RMPs were broadly distributed, with frequent representation of “Other agents affecting metabolism,” “Antineoplastic agents,” and “Agents affecting central nervous system” (Table 1). Among the MedDRA-classified IPRs, “Neoplasms benign, malignant and unspecified (incl cysts and polyps),” “Injury, poisoning and procedural complications,” and “Infections and infestations” were the most common risk types (Table 2).

**Table 1:** Background information on risk management plans

	N
Risk management plans	330
Therapeutic categories	
Other agents affecting metabolism	64
Antineoplastic agents	47
Agents affecting central nervous system	33
Biological preparations	26
Agents affecting digestive organs	22
Hormone preparations (including antihormone preparations)	20
Chemotherapeutics	20
Cardiovascular agents	18
Antibiotic preparations	10

Only categories cited at least 10 times in risk management plans were included

**Table 2:** Background information on important potential risks

	N
Important potential risks	772
Risk type (MedDRA System Organ Class)	
Neoplasms benign, malignant and unspecified (incl cysts and polyps)	81
Injury, poisoning and procedural complications	59
Infections and infestations	55
Immune system disorders	52
Cardiac disorders	51
Nervous system disorders	50
Vascular disorders	43
General disorders and administration site conditions	37
Gastrointestinal disorders	34
Investigations	32

Only MedDRA System Organ Class categories containing at least 30 important potential risks were included

Of the 772 IPRs, 134 (17.4%; 95% CI, 14.8%–20.2%) were not documented in the PI. Validation in a random subset of 60 IPRs revealed high agreement between the RMP-based classification and direct PI review (98.3%; Cohen’s κ = 0.96; sensitivity = 94.1%; specificity = 100%). The misclassification-corrected estimate was 18.4%, essentially unchanged from the observed proportion.

**Fig. 2:**
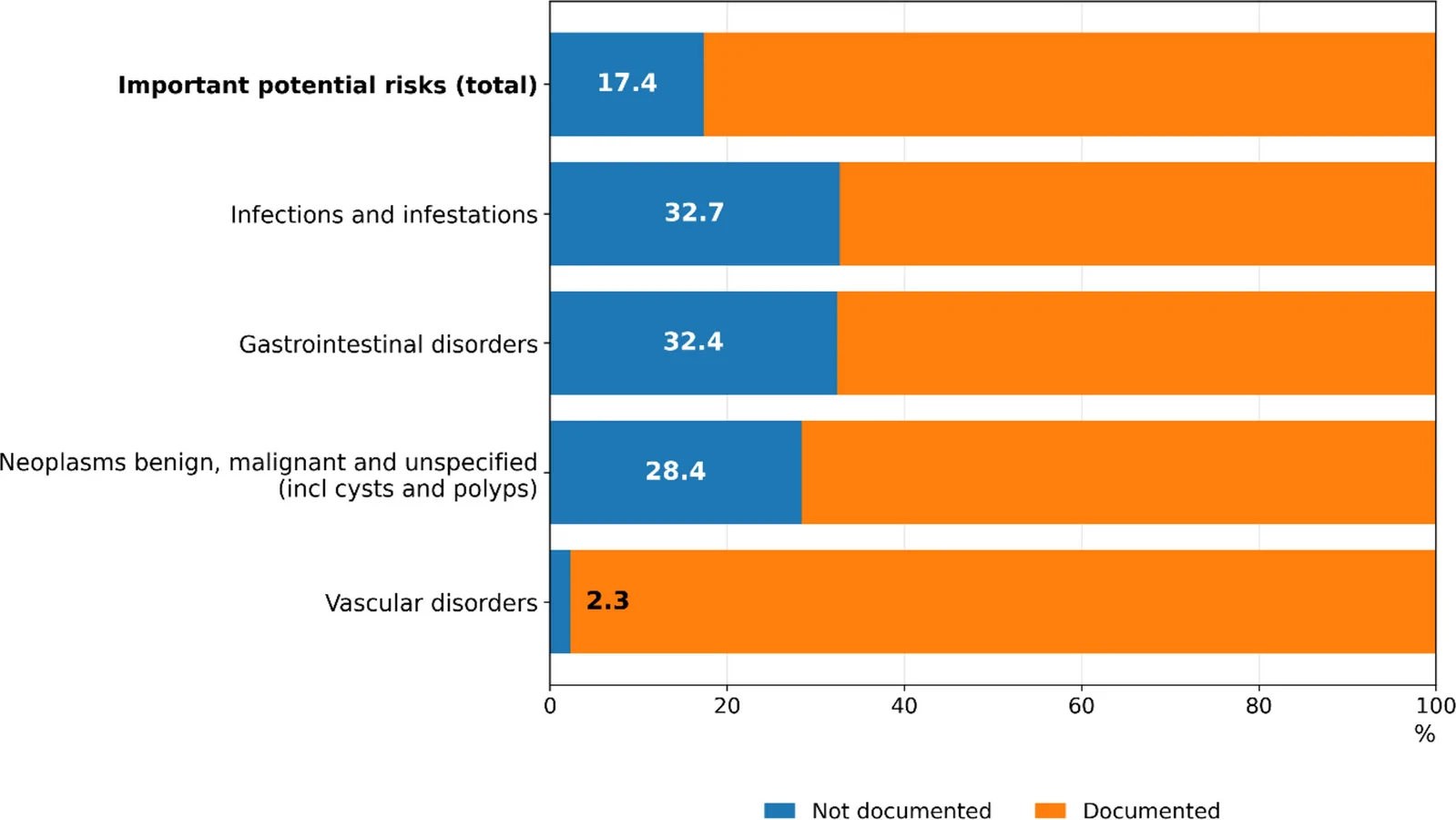
Rates of non-documentation of important potential risks in the package insert: Overall and selected risk types highlighted in the Results

In the descriptive analyses by risk type, among MedDRA System Organ Class categories containing at least 30 IPRs, relatively high proportions of non-PI documentation were observed for “Infections and infestations” (32.7%), “Gastrointestinal disorders” (32.4%), and “Neoplasms benign, malignant and unspecified (incl cysts and polyps)” (28.4%). In contrast, a relatively low proportion was observed for “Vascular disorders” (2.3%). All IPRs classified under “Pregnancy, puerperium and perinatal conditions,” “Reproductive system and breast disorders,” and “Congenital, familial and genetic disorders” were documented in the PI (Table 3). The main trends are also illustrated in Fig. 2. For the 134 IPRs not documented in the PI, the reasons for non-documentation included unclear manifestation risk, absence of domestic cases, a lack of precautionary content, or non-inclusion in the PIs of reference products.

**Table 3:** Important potential risks not documented in the package insert

	N	Not documented in the package insert	%
Important potential risk	**772**	**134**	**17.4**
Risk type			
Neoplasms benign, malignant and unspecified (incl cysts and polyps)	81	23	28.4
Injury, poisoning and procedural complications	59	8	13.6
Infections and infestations	55	18	32.7
Immune system disorders	52	6	11.5
Cardiac disorders	51	14	27.5
Nervous system disorders	50	10	20.0
Vascular disorders	43	1	2.3
General disorders and administration site conditions	37	5	13.5
Gastrointestinal disorders	34	11	32.4
Investigations	32	7	21.9
Eye disorders	29	3	10.3
Skin and subcutaneous tissue disorders	28	2	7.1
Blood and lymphatic system disorders	27	0	0.0
Renal and urinary disorders	24	2	8.3
Hepatobiliary disorders	22	4	18.2
Respiratory, thoracic and mediastinal disorders	22	3	13.6
Metabolism and nutrition disorders	21	3	14.3
Psychiatric disorders	18	3	16.7
Musculoskeletal and connective tissue disorders	16	3	18.8
Endocrine disorders	12	1	8.3
Pregnancy, puerperium and perinatal conditions	11	0	0.0
Reproductive system and breast disorders	6	0	0.0
Surgical and medical procedures	5	5	100.0
Congenital, familial and genetic disorders	4	0	0.0
Safety and efficacy in specific populations	33	2	6.1

Risk types were primarily classified according to MedDRA System Organ Classes; “Safety and efficacy in specific populations” was treated as a separate non-MedDRA category. The 95% confidence interval for the overall non-documentation proportion was 14.8%–20.2%

Among the 638 IPRs documented in the PI, information on the PI section was available for 581 IPRs. The most common section was “11. ADVERSE REACTIONS,” followed by “8. IMPORTANT PRECAUTIONS,” “9. PRECAUTIONS CONCERNING PATIENTS WITH SPECIFIC BACKGROUNDS,” and “15. OTHER PRECAUTIONS” (Table 4). Among IPRs documented in “1. WARNINGS,” “Neoplasms benign, malignant and unspecified (incl cysts and polyps)” was the most frequent risk type. In “2. CONTRAINDICATIONS,” “Immune system disorders” and “Neoplasms benign, malignant and unspecified (incl cysts and polyps)” were the most frequent risk types.

**Table 4:** Package insert sections for important potential risks

	N	%
Important potential risks with available package insert section information	581	100.0
Sections of the package insert		
Introductory section	8	1.4
1. WARNINGS	20	3.4
2. CONTRAINDICATIONS	42	7.2
4. INDICATIONS	1	0.2
5. PRECAUTIONS CONCERNING INDICATIONS	8	1.4
6. DOSAGE AND ADMINISTRATION	3	0.5
7. PRECAUTIONS CONCERNING DOSAGE AND ADMINISTRATION	28	4.8
8. IMPORTANT PRECAUTIONS	184	31.7
9. PRECAUTIONS CONCERNING PATIENTS WITH SPECIFIC BACKGROUNDS	122	21.0
10. INTERACTIONS	37	6.4
11. ADVERSE REACTIONS	247	42.5
11.1 Clinically Significant Adverse Reactions*	*120*	*51.3*
11.2 Other Adverse Reactions*	*119*	*50.9*
12. INFLUENCE ON LABORATORY TESTS	1	0.2
13. OVERDOSAGE	12	2.1
14. PRECAUTIONS CONCERNING USE	8	1.4
15. OTHER PRECAUTIONS	102	17.6
16. PHARMACOKINETICS	6	1.0
17. CLINICAL STUDIES	3	0.5
20. PRECAUTIONS FOR HANDLING	2	0.3

Among the 638 IPRs documented in the PI, information on the PI section was available for 581 IPRs. For Section 11, “Adverse Reactions,” subsection-level analyses were restricted to the 234 IPRs for which subsection information was available. Only descriptive summaries are presented, as no hypothesis testing was performed. An important potential risk can appear in multiple package insert sections or sub-sections; thus, the totals can exceed 100%

## Discussion

In this study, 17.4% of IPRs were not documented in the PI, in line with the rate of 14.0% in our previous study on products for which RMPs had been completed [6]. According to the RMPs, the reasons for not including certain IPRs in the PI often included unclear manifestation risks. For example, in the descriptive subgroup analysis, a relatively high proportion of non-PI documentation was observed for “Neoplasms benign, malignant and unspecified (incl cysts and polyps).” Malignancies tend to require a long latency period for manifestation [18, 19], resulting in few reported cases and insufficient accumulation of safety data. Similarly, “Infections and infestations” often involve complex causality assessments because of confounding factors such as the patient’s underlying condition or coincidental outbreaks [20], making it difficult to clearly attribute the risk to the drug. Risk types characterized by long latency periods or unclear causal relationships with the drug are thus presumed less likely to appear in the PI.

IPRs documented in the PI were also included in sections beyond “11. ADVERSE REACTIONS,” with frequent entries in “8. IMPORTANT PRECAUTIONS,” “9. PRECAUTIONS CONCERNING PATIENTS WITH SPECIFIC BACKGROUNDS,” and “15. OTHER PRECAUTIONS”. These sections allow both the IPRs themselves and the underlying information that led to their designation, such as clinical or nonclinical findings alongside other underlying evidence, to be highlighted in ways that support clinical awareness. Therefore, although some IPRs were functionally addressed in the PI, the visibility of those not documented in the PI relied solely on the RMP. For healthcare professionals, the absence of an IPR from the PI does not necessarily indicate the absence of an unresolved potential risk; therefore, RMP-derived information should be considered, where available, in clinical decision-making.

Against this backdrop, the Japanese regulatory framework poses a distinct challenge: most RMPs are terminated at the time of re-examination, and the associated materials are removed from the PMDA website [6, 21]. Consequently, once an RMP ends, IPRs not documented in the PI can no longer be readily identified by healthcare professionals through either the PI or the PMDA RMP source, creating a gap in information access. However, it might not be appropriate to always include all unresolved IPRs in the PI, as overloading prescribers with speculative or weakly evidenced risks could undermine the practical utility of the PI. Internationally, unresolved potential risks are often managed within the RMP rather than the product labeling. For example, in the EU, RMPs are maintained throughout the entire product lifecycle, allowing unresolved risks to remain visible even if not included in the Summary of Product Characteristics [22, 23]. In addition, the United States Prescribing Information is generally limited to well-established safety information, whereas serious safety concerns might trigger the imposition of a Risk Evaluation and Mitigation Strategy [24]. By contrast, in Japan, termination of most RMPs at re-examination limits the continued visibility of unresolved IPRs not documented in the PI. This structural difference illustrates that the key challenge is not to mandate PI inclusion of all IPRs; rather, the practice of ending RMPs at re-examination should be reconsidered to ensure continued visibility of unresolved safety concerns.

On this point, the subcommittee of the Japanese authority issued a report in January 2025 titled, “Summary of the Revisions to the Pharmaceutical and Medical Device Act and Related Systems” [25], which emphasized the need to revise the law to make the submission of RMPs and the implementation of risk management activities a legal obligation. The report highlighted a flaw in the current law, specifically that safety measures based on RMPs are uniformly terminated when post-marketing re-examination conditions are lifted. Instead of requiring the submission and implementation of an RMP as an approval condition, it proposed mandating them by law, permitting more flexible and timely responses to emerging safety concerns. In line with this recommendation, the Act on Securing Quality, Efficacy and Safety of Products Including Pharmaceuticals and Medical Devices was revised in May 2025 [26]. Although no concrete measures were specified regarding the handling of RMPs at the conclusion of the re-examination period, the proposal aligns with the issues raised in our study and represents a promising regulatory development. We hope that this will lead to more flexible options, such as continuing RMPs beyond the re-examination period when unresolved IPRs remain or reactivating RMPs in response to new safety concerns. Close monitoring of the implementation of this framework in practice is necessary.

This study has several limitations. It did not evaluate downstream safety outcomes; therefore, its implications concern access to and communication of unresolved risk information rather than a demonstrated increase in adverse events. Given the exploratory and descriptive nature of the subgroup analysis, the observed differences among risk types should be interpreted cautiously and regarded as hypothesis-generating. In this study, the presence or absence of PI documentation for IPRs was inferred from whether the RMP specified “inclusion in the PI” as a risk minimization activity, rather than determined by direct review of the PI text. This approach was adopted to avoid subjective judgment, as visual, medically based assessments can vary among reviewers and introduce inconsistency [15]. Nonetheless, discrepancies might exist between the actual PI content and the stated risk minimization plans in the RMP. To address this concern, we validated the classification in a random subset of 60 IPRs, observing extremely high agreement (κ = 0.96), and the misclassification-corrected estimate was essentially unchanged from the observed proportion, supporting the robustness of our findings. However, some residual misclassification may persist if the actual PI content differed from the risk minimization activities stated in the RMP. In addition, the exclusion of RMPs that were revised or removed during the one-year period before the data cutoff may have introduced selection bias; therefore, the findings may not fully represent all RMPs that were active at the initial reference date. This study was not designed to disentangle the determinants of PI inclusion, and potential influences, including risk characteristics and regulatory precedent, warrant future study.

## Conclusion

This study revealed that some IPRs in RMPs were not documented in the PI. Because most RMPs in Japan currently end at the time of re-examination, undocumented IPRs can no longer be readily identified by healthcare professionals through either the PI or the PMDA RMP source, limiting transparency. Maintaining visibility of such risks may help support informed clinical decisions and sustained regulatory communication. To address this issue, a framework that allows RMPs to be continued beyond the re-examination period when necessary will be important.

## Data Availability

Data will be made available upon reasonable request.
